# Supplementary material for: Patterns of Intron Gain and Loss in Fungi
Source: PLoS Biol. 2004 Nov 30;2(12):e422. doi: 10.1371/journal.pbio.0020422 (PMC532390; doi:10.1371/journal.pbio.0020422)
Supplement: Table S1 — Also available at http://genes.mit.edu/NielsenEtAl/. (4.3 MB ZIP). [file pbio.0020422.st001.zip › NielsenEtAl/html/16.html]

AN6179.1.NCU03309.1.MG09887.1.FG01118.1


```
 CLUSTAL W (1.82) Multiple Sequence Alignments - Introns Inserted


Sequence 1: AN6179.1	81 aa
Sequence 2: NCU03309.1	78 aa
Sequence 3: MG09887.1	63 aa
Sequence 4: FG01118.1	80 aa
Alignment Length: 81 aa
Number Identitical Residues: 48 aa
Alignment Score (without introns) 2082


MG09887.1 	----~----------MDIESDYK0VSQIKEKVEEKEGIPPVQQRLIFGGKQM2VDDKTAA
NCU03309.1	MQIK2VRTLTGKEIELDIEPDTK0VAHIKEKVEEKEGIPPVQQRLIFGGKQM2VDDKTAT
FG01118.1 	MLIK2VRTLTGKEIELDIESDYK0VSQIKEKVEEKEGIPPVQQRLIHGGKQM2TDDKTAA
AN6179.1  	MLIK2VRTLTGKEIELDIEPDYK0VSRIKERVEEKEGIPPVQQRLIFGGKQM2ADDKTAQ
          	   .   : :... .:***.* * *::***:***************.***** .***** 

MG09887.1 	DYQLEGGSTLHLVLALRGGC----
NCU03309.1	DYQLEGGATLHLVLALRGGRW---
FG01118.1 	EYNLVAGDTLHLVLALRGGRWLS-
AN6179.1  	DYNLEGGATLHLVLALRGGCAALQ
          	:*:* .* ***********    .
```
